# A Systematic Review of Benefits and Risks of Fetal Surgery for Congenital Cardiac Defects Such as Pulmonary Valve Stenosis and Critical Aortic Stenosis

**DOI:** 10.21470/1678-9741-2022-0273

**Published:** 2023

**Authors:** Ana Maria Bicudo Diniz, Paulo Henrique Manso, Marcelo Volpon Santos, Alfredo José Rodrigues, Lourenço Sbragia

**Affiliations:** 1 Division of Pediatric Surgery, Department of Surgery and Anatomy, Faculdade de Medicina de Ribeirão Preto, Universidade de São Paulo, Ribeirão Preto, São Paulo, Brazil; 2 Division of Pediatric Cardiology, Department of Pediatrics, Faculdade de Medicina de Ribeirão Preto, Universidade de São Paulo, Ribeirão Preto, São Paulo, Brazil; 3 Division of Pediatric Neurosurgery, Department of Surgery and Anatomy, Faculdade de Medicina de Ribeirão Preto, Universidade de São Paulo, Ribeirão Preto, São Paulo, Brazil; 4 Division of Cardiothoracic Surgery, Faculdade de Medicina de Ribeirão Preto, Universidade de São Paulo, Ribeirão Preto, São Paulo, Brazil

**Keywords:** Aortic Valve Stenosis, Hypoplastic Left Heart Syndrome, Heart Defects, Congenital Pulmonary Valve Stenosis, Fetal Development

## Abstract

**Introduction:**

Congenital heart diseases (CHDs) constitute the most prevalent congenital pathology, and they are a consequence of structural and functional abnormalities during fetal development. The etiology of CHD involves the interaction of genetic and environmental factors. Fetal cardiac surgery aims at preventing natural pathways of CHD *in utero*, mitigating progression to more complex abnormalities. The goal of this review was to demonstrate the benefits and risks of fetal interventions in the two most prevalent CHDs, pulmonary stenosis and pulmonary atresia with an intact ventricular septum, but also critical aortic stenosis and hypoplastic left heart syndrome.

**Methods:**

Original and relevant articles were selected by meta-aggregation to perform a qualitative analysis of fetal cardiac interventions for pulmonary stenosis and critical aortic stenosis. The Joanna Briggs Institute’s Qualitative Assessment and Review Instrument (or JBI-QARI) was used for data quality appraisal.

**Results:**

Of 61 potential articles, 13 were selected, and nine were finally included. Discussion: The present review demonstrated that fetal cardiac surgery increases right ventricular growth and hemodynamic flow in pulmonary stenosis, whereas in critical aortic stenosis it enables growth of the left ventricle and increases left ventricular pressure. However, it has a high complication rate, along with considerable morbidity and mortality.

**Conclusion:**

The benefits of fetal cardiac surgery for pulmonary stenosis and critical aortic stenosis are well-described in the literature; however, there is a significant risk of complications which can be reduced by the surgeon’s technical expertise and well-structured hospital facilities.

## INTRODUCTION

The term congenital heart disease (CHD) refers to defects in the structure and function of the heart and its blood vessels. It is a public health issue and the most common congenital disease, with an estimated prevalence of eight to 11 per 1,000 live births^[1]^. In 2010, 25,757 new children were born with CHD in Brazil. However, data published in the medical literature are still conflicting because of CHD subnotification^[2]^.

About 9/1,000 of births have some form of CHD. Congenital diseases are surely a social problem that impacts the quality of life of patients and their families, and also contributes to fetal and infant morbidity and mortality, reduces life expectancy, as well as the family’s well-being, and increases cost of living due to special prenatal care, medical and social educational services, let alone the psychological burden^[1]^.

The etiology of CHD is not fully understood. However, it is known that interaction of genetic and environmental factors can ultimately lead to CHD^[3]^. For instance, maternal conditions like alcohol abuse, use of prescribed medications, diabetes mellitus, and obesity increase the likelihood of CHD. In addition, many genetic and chromosomal abnormalities, including Down syndrome, Turner syndrome, DiGeorge syndrome, and maternal viral infections, such as Rubella, are related to CHD^[3,4]^.

Some authors claim that certain complex heart defects may result from the progression of fetal heart responses to simple injuries^[5]^. Therefore, the objective of fetal interventions is to prevent the consequences of this first-hit lesion which give rise to complex secondary ones. Fetal surgery can ameliorate cardiac structure and function, and thus change the intrauterine course of CHD^[6]^.

The present article has focused on CHDs whose treatment with fetal cardiac interventions (FCI) have been described in the medical literature — pulmonary stenosis (PS), pulmonary atresia with intact ventricular septum (PAIVS), critical aortic stenosis (CAS), and hypoplastic left heart syndrome (HLHS) —, providing a qualitative interpretation of the results of fetal cardiac surgery in humans.

### Overview of Pulmonary Stenosis and Pulmonary Atresia with Intact Ventricular Septum

PS has an incidence of 0.6 to 0.8 per 1,000 live births, and PAIVS occurs in 0.083 per 1,000 live births^[7]^.

Prenatal detection of these conditions is actually low. Just 37% of critical PS and 60% of PAIVS are diagnosed in prenatal care^[8]^. Prenatal diagnosis is usually made when the right ventricle (RV) is hypertrophic or hyperplastic; or, in some cases, when ventriculocoronary connections can be seen^[9]^. Both lesions promote an obstruction to right ventricular outflow, leading to right ventricular dysfunction^[10]^.

Depending upon the gestational age of pulmonary valve obstruction and its repercussion, this obstruction may lead to a hypoplastic or normal sized but dysfunctional right ventricle. Therefore, fetal interventions intend to relieve right ventricular outflow tract (RVOT) obstruction and avoid right ventricular malfunction or hypoplasia^[11,12]^.

FCI of PS and PAIVS is performed under ultrasound guidance and maternal general anesthesia^[13]^; initially, a 19-gauge cannula and stylet are introduced into the RV through the maternal abdomen, uterine wall, and fetal chest. Access to the RVOT should be reached via a subcostal route or intercostal space behind the sternum. A stylet or a 22-gauge Chiba needle punches the RV. Then, a 0,014” wire and a coronary balloon are placed and inflated across the valve^[14]^.

The use of maternal general anesthesia with inhalational agents is the surgical choice because it improves uterine relaxation and facilitates fetal manipulation and positioning. Afterwards, the fetus is medicated with intramuscularly administered fentanyl, atropine, and pancuronium^[13]^. The technical success of this FCI is set by color Doppler examination that shows flow through the pulmonary valve^[12,14]^.

According to literature data^[13]^, fetuses who underwent FCI with balloon dilatation have better prognosis than controls with PAIVS not operated *in utero*. They had significant growth of the PS annulus and tricuspid valve, and also successful univentricular outcomes after birth, from midgestation to late gestation. Operated newborns had right ventricular growth and moderate right heart hypoplasia^[13]^, which was much more similar to a biventricular outcome^[14]^. Reports also indicate that FCI for the pulmonary valve has limited applications; only a tiny subset of fetuses with PS and PAIVS are eligible for intervention; also, there is also a lack of criteria regarding the acceptable size of right ventricular circulation after pulmonary valve interventions^[13,15]^.

### Overview of Critical Aortic Stenosis

CAS is a dynamic and progressive malformation with variable hypoplasia on the left heart^[16]^, which is a hallmark of this pathology; thus, the RV works as the systemic pump (univentricular palliation), leading to insufficiency of the systemic circulation and constituting a surgical challenge with significant perioperative mortality and long-term morbidity^[17]^.

The development of HLHS secondary to CAS occurs at midgestation, specifically when there is left ventricular (LV) dilation or dysfunction and retrograde flow into the transverse aortic arch. In these cases, early intervention can protect the fetus from HLHS, enabling biventricular circulation (BV) and improving short-term and long-term mortality and morbidity^[17]^. For this subtype of severe aortic stenosis, fetal aortic valvuloplasty (FAV) is indicated, which is the most common FCI performed. This intervention improves left heart hemodynamics and growth of aortic and mitral valves^[17,18]^.

McElhinney et al.^[18]^ usually obtain preoperative measurements of the aortic annulus and the distance between valvular hinge points using echocardiography. Then, if external maneuvers are unsuccessful, a limited laparotomy is performed for uterine manipulation and imaging, allowing for proper fetus positioning. The fetus receives an intramuscular anesthetic and muscle relaxant for catheterization. A low-profile over-the-wire coronary angioplasty catheter with a balloon diameter is chosen based on the measurement of the aortic annulus. The balloon catheter is set up with a foppy-tipped guidewire. Under ultrasound guidance, the wire/catheter is introduced into the fetal chest wall directed to the LV epicardium. The catheter is manipulated until it fits into the valve. Lastly, the balloon is inflated, and all instruments are withdrawn. The procedure is deemed successful when at least one balloon inflation across the aortic valve is achieved^[19]^.

## METHODS

This review has utilized the Joanna Briggs Institute (JBI) method for meta-aggregation and evaluative processes, particularly the JBI's Qualitative Assessment and Review Instrument (JBI -QARI) system for quality appraisal^[20]^, which creates a synthesis between studies for improvement of practice. Meta-aggregation was chosen to qualitatively evaluate Fetal cardiac interventions in humans from a global perspective and their implications on clinical practice and research. As per the JBI method, the data sources’ guiding question was “Does fetal cardiac surgery benefit children with congenital cardiac defects?”. This article also incorporated the revised PICO mnemonic, which refers to population (P), intervention (I), comparators (C), and outcomes (O) for qualitative data analysis. Inclusion criteria were population diagnosed with congenital cardiac disease at prenatal care, and that underwent Fetal cardiac intervention; clear outcome data; and published in English. PubMed database was used for data extraction, searching for the following synonyms, keywords, and descriptors: “fetal cardiac disease intervention”, “cardiac fetal surgery”, “fetal diagnosis cardiac malformation”, “fetal interventions for congenital heart disease”, “pulmonary stenosis fetal intervention”, “critical aortic stenosis fetal intervention”, “fetal intervention”, “fetal surgery”, “fetal pulmonary stenosis”, “fetal pulmonary atresia with intact ventricular septum”. Exclusion criteria were nondiscriminatory data between distinct cardiac fetal diseases; nondiscriminatory data between distinct cardiac fetal interventions; articles whose title and abstract did not answer the data source question; articles whose full text did not answer the data source question; experimental (animal) studies; case reports, meta-analyses, and reviews; and articles not published in English.

## RESULTS

**Fig. 1:**
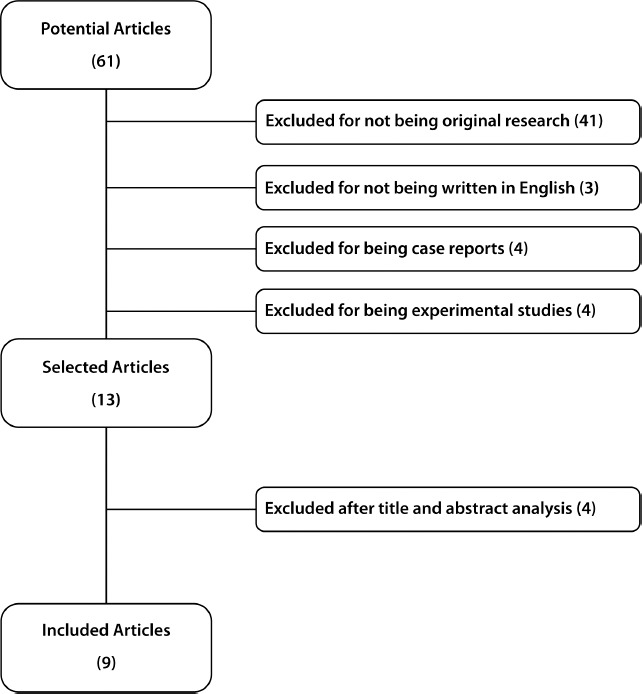
PRISMA chart of the study design and included manuscripts.

The database search selected 61 potential articles. Of these, 41 were excluded for not being original research, three were excluded for not being written in English, four were case reports, and four were excluded for being experimental research. The remaining 13 articles were further assessed, and four were excluded after the title, abstract, or full text were read. Finally, nine studies that met the selection criteria were included. This process is demonstrated in the PRISMA chart depicted in Figure 1 and specified in Tables 1 and 2.

**Table 1:** Joanna Briggs Institute's Qualitative Assessment and Review Instrument (JBI-QARI) of included articles.

JBI-QARI	Study 1	Study 2	Study 3	Study 4	Study 5	Study 6	Study 7	Study 8	Study 9
	Tworetzky W et al.^[14]^	McElhinney DB et al.^[18]^	Arzt W et al.^[25]^	Gómez Montes E et al.^[22]^	Pedra SR et al.^[22]^	Freud LR et al.^[27]^	Cruz-Leini M et al.^[27]^	Hogan WJ et al.^[22]^	Patel ND et al.^[28]^
Is there congruity between the stated philosophical perspective and the research methodology?	Yes	Yes	Yes	Yes	Yes	Yes	Yes	Yes	Yes
Is there congruity between the research methodology and the research question or objectives?	Yes	Yes	Yes	Yes	Yes	Yes	Yes	Yes	Yes
Is there congruity between the research methodology and the methods used to collect data?	Yes	Yes	Yes	Yes	Yes	Yes	Yes	Yes	Yes
Is there congruity between the research methodology and the representation and analysis of data?	Yes	Yes	Yes	Yes	Yes	Yes	Yes	Yes	Yes
Is there congruity between the research methodology and the interpretation of results?	Yes	Yes	Yes	Yes	Yes	Yes	Yes	Yes	Yes
Is there a statement locating the researcher culturally or theoretically?	No	No	No	No	No	No	No	No	No
Is the influence of the researcher on the research, and vice-versa, addressed?	No	No	No	No	No	No	No	No	No
Are participants, and their voices, adequately represented?	Yes	Yes	Yes	Yes	Unclear	Yes	Yes	Yes	Yes
Is the research ethical according to current criteria or, for recent studies, and is there evidence of ethical approval by an appropriate body?	Yes	Yes	Yes	Yes	Yes	Yes	Yes	Yes	Yes
Do the conclusions drawn in the research report flow from the analysis, or interpretation, of the data?	Yes	Yes	Yes	Yes	Yes	Yes	Yes	Yes	Yes

**Table 2:** Summary of fetal cardiac interventions (FCIs) and successful biventricular outcomes.

Article	Year	CHD	Attempted FCI	Successful procedures	Post procedural deaths	Failed biventricular outcome	Successful biventricular outcome
Tworetzky W et al.^[14]^	2009	PS/PAIVS	10	6	1	6	4
McElhinney DB et al.^[18]^	2009	CAS/HLHS	70	52	11	42	17
Arzt W et al.^[25]^	2011	CAS/HLHS	24	16	3	10	11
Gómez Montes E et al.^[22]^	2012	PS/PAIVS	4	4	2	1	1
Pedra SR et al.^[21]^	2013	CAS/HLHS	18	17	7	3	6
Freud LR et al.^[27]^	2014	CAS/HLHS	100	77	12	57	31
Cruz-Lemini M et al.^[26]^	2018	CAS/HLHS	9	9	3	2	4
Hogan WJ et al.^[22]^	2020	PS/PAIVS	58	41	9	11	32
Patel ND et al.^[28]^	2020	CAS/HLHS	108	90	47	25	34

CAS=critical aortic stenosis; CHD=congenital heart disease; HLHS=hypoplastic left heart syndrome; PAIVS=pulmonary atresia with intact ventricular septum; PS=pulmonary stenosis

## DISCUSSION

### Pulmonary Stenosis and Pulmonary Atresia with Intact Ventricular Septum

A comprehensive description of pulmonary valvuloplasty for membranous pulmonary atresia with traceable pulmonary valve membrane or cups, intact ventricular septal defect, right heart hypoplasia, and tricuspid valve annulus was published by Gómez Montes et al.^[22]^. They have also shown that this procedure increases right ventricular growth and hemodynamic flow^[22]^.

Hoganet al.^[23]^ conducted an international FCI registry for PS/PAIVS comprising data from 84 patients of 14 international institutions, evaluating the potential benefit of pulmonary valvuloplasty and transatrial fetal atrial septoplasty. Their study^[23]^ included PAIVS and critical PS between 2001 and 2018. Fifty-eight maternal-fetal dyads underwent FCI, of whom 41 were successful, 15 unsuccessful, and two unknowns. There were nine fetal deaths related to the procedure. Also, no maternal complications were reported. Fetuses that were operated on had larger tricuspid valve annuli and greater degrees of tricuspid regurgitation than those that did not undergo FCI. The authors concluded that, although more studies are needed to confirm the results of FCI on tricuspid valves, a sensible difference could be observed between operated and non-operated fetuses; on the other hand, complication rates associated with FCI are high, and occurred in 32/58 of the cases. The most common was pericardial effusion requiring drainage in 28/58 fetuses and bradycardia requiring treatment in 21/58 fetuses.

Tworetzky et al.^[24]^ described their experience with FCI for PAIVS patients who were submitted to intrauterine balloon dilation of the pulmonary valve during midgestation. FCI-submitted fetuses were compared with non-operated counterparts in late gestation. Ten FCIs were reported; the first four interventions were unsuccessful, while the latter six were successful. Successful interventions were more frequent in older fetuses (between 23–28 weeks) than in younger ones (between 21–24 weeks), which, however, was not statistically significant, probably due to the limited number of cases. Of the 10 FCIs, nine were born full-term. They all underwent neonatal interventions, such as pulmonary balloon valvuloplasty (six patients) and surgical procedures (eight patients), including bidirectional Glenn and Fontan procedures, systemic-to-pulmonary artery shunt, and RVOT patch. Also, they were alive on a 0.5 to 5.8-year follow-up. The comparison with the group of patients not submitted (15 patients) to FCI showed that the successful intervention group had a pivotal right heart growth from midgestation to late gestation.

### Critical Aortic Stenosis with Hypoplastic Left Heart Syndrome

Pedra et al.^[21]^ performed a study that analyzed an initial FCI performed in 22 fetuses of a Brazilian institution, of which 13 had CAS, four had HLHS, and five had intact interatrial septum or small patent foramen ovale. Procedures were successful in 20 of 22 fetuses (91%) with only one fetal death. These results are comparable with similar international reports^[21]^.

McElhinney et al.^[18]^ performed aortic valvuloplasty on 70 fetuses diagnosed with severe aortic stenosis and HLHS at 23 weeks of gestation. FCI was technically successful in 74% of fetuses. Comparing with non-operated fetuses, the authors clearly observed growth of mitral and aortic valves, but no growth of the left ventricle at the end of pregnancy. However, larger left ventricles and higher LV pressures were present in 21% of patients after birth. They concluded that FCI allows for the possibility of biventricular outflow postnatally, which is unlikely in non-FCI patients^[18]^.

Arzt et al.^[25]^ performed aortic valvuloplasty for severe aortic stenosis with HLHS on 24 midgestation fetuses, of whom 15 were successfully treated and liveborn. Of these, 10 had BV postnatally. Their article emphasizes that safety and success of FCI depend on patient selection and experience of the interventional team, showing that successful performances improved from 69.9% to 78.6% after an initial learning curve^[25]^.

Cruz-Lemini et al.^[26]^ reported the results of HLHS and FAV in Mexico, a country with suboptimal postnatal management, similar to Brazil. Nine fetuses with HLHS were operated, all technically successful. There were three fetal deaths (one intraoperative death related to bradycardia and two after 72 hours of FCI). The survival group was followed up from 24 months to six years of age; long-term survival was 44%, which is comparable to the medical literature, and one patient was later submitted to palliative surgery. Regarding HLHS outcomes, although the liveborn rate was 86%, surgical palliation was attempted in only three cases; thus, overall survival was 4%, despite 50% to 90% for the first stage of surgical palliation.

Freud et al.^[27]^ published a clinical trial held in Boston Children’s Hospital of 100 patients with severe aortic stenosis that underwent FAV at midgestation. About 88% were liveborn, 38% with BV. Aortic and mitral valve sizes were more prominent in BV patients at birth; also, in this group, there was no mortality^[28]^. This overview is extremely different from the reality exposed in the Mexican publication, certainly due to lack of resources, as well as availability of skilled multidisciplinary teams^[27]^.

Patel et al.^[28]^ evaluated maternal-fetal outcomes after FAV in cases of severe HLHS. Of 108 fetuses that underwent FCI, 83% of interventions were technically successful. Intraoperative complications were found in 48%, including bradycardia and pericardial or pleural effusions. Large cannula sizes (< 19 gauge) were related to higher chances of pericardial effusion as compared to 17 and 18-gauge cannula. Eighty-one babies were born alive: thus, 34 developed BV. In addition, higher complication rates and fetal mortality were associated with multiple cardiac punctures, which, despite being risky, are frequently successful^[18]^.

### Relevance For Clinical Practice

The present review has the importance of evaluating advances and challenges of fetal heart interventions worldwide and especially in low-income countries such as Brazil. In fact, very little original research has shown the reality of FCI in low-income countries, where FCI are mostly experimental and limited to a few medical services. Therefore, the viability, indication, and risks of FCI has particularities on these locations.

Even in high-income countries, uniform criteria for FCI are not well-established; such procedures are usually indicated once there is a substantial risk of morbidity and mortality for the fetus, so that unnecessary FCI should be avoided. Also, there are variable results of risks and benefits of fetal pulmonary and aortic valvuloplasty, suggesting that clinical outcomes are related to multiple factors such as experience of the surgical team, inclusion criteria, and available resources. In spite of its risks, balloon dilation *in utero* seems to change the natural history of PAIVS.

Concerning fetal therapy for PAIVS/PS, there is a relative lack of original articles as compared to CAS. Current findings suggest that surgical treatment is promising, since it promotes right heart growth. However, considerable rates of procedure-related fetal deaths and mobility still remain; thus, uniform and well-established criteria are also needed. Moreover, robust data comparing FCI and postnatal interventions are required to precisely analyze the best timing for surgery.

## CONCLUSION

Although there are many reports about the benefits of intrauterine valvuloplasty, these procedures are still associated with a high risk of complications and mortality. Thus, prior to a more widespread utilization of FCI, further refinements are essential, such as minimizing surgical invasion, development of experienced clinical staf, and provision of well-equipped hospitals.
